# Long–Term Disease Control After Allogeneic Hematopoietic Stem Cell Transplantation in Primary Cutaneous T–Cell Lymphoma; Results From a Single Institution Analysis

**DOI:** 10.3389/fmed.2020.00290

**Published:** 2020-06-25

**Authors:** Florentia Dimitriou, Urs Schanz, Gayathri Nair, Susanne Kimeswenger, Marie-Charlotte Brüggen, Wolfram Hoetzenecker, Lars E. French, Reinhard Dummer, Antonio Cozzio, Emmanuella Guenova

**Affiliations:** ^1^Department of Dermatology, University Hospital Zurich, Zurich, Switzerland; ^2^Faculty of Medicine, University of Zurich, Zurich, Switzerland; ^3^Department of Medical Oncology and Hematology, University Hospital Zurich, Zurich, Switzerland; ^4^Department of Dermatology, Kepler University Hospital Linz, Linz, Austria; ^5^Department of Soft Matter Physics, Institute for Experimental Physics, Johannes Kepler University, Linz, Austria; ^6^Department of Dermatology, University Hospital Munich (LMU), Munich, Germany; ^7^Department of Dermatology, Kantonsspital St Gallen, St Gallen, Switzerland; ^8^Lausanne University Hospital (CHUV) and Faculty of Biology and Medicine, University of Lausanne, Lausanne, Switzerland

**Keywords:** allogeneic stem cell transplantation, cutaneous T-cell lymphoma, Sézary syndrome, mycosis fungoides, interferon alpha-2a

## Abstract

**Background:** Allogeneic hematopoietic stem cell transplantation (alloHSCT) has been proposed as curative approach for advanced cutaneous T–cell lymphomas (CTCL). Currently, there is no established consensus for the management of disease relapse after alloHSCT.

**Results:** Ten patients, previously treated with multiple lines of systemic treatment, received alloHSCT. Six patients had achieved partial response (PR, *N* = 5) and complete response (CR, *N* = 1) prior to HSCT. Post—HSCT, seven patients (*N* = 7) relapsed after a median time of 3.3 months (0.5–7.4 months) and were subsequently treated with radiotherapy (RT, *N* = 1), RT and adoptive T-cell transfer with EBV specific cells (*N* = 1), R-CHOP (*N* = 1) and interferon alpha−2a combined either with donor lymphocyte infusion (*N* = 1) or with brentuximab—vedotin (*N* = 1). One patient (*N* = 1) achieved PR only after reducing the immunosuppression. Two patients relapsed again and received interferon alpha−2a and brentuximab—vedotin, respectively. After a median follow-up time of 12.6 months (3.5–73.7 months) six patients were alive (60%) and four had deceased, three (*N* = 3) due to CTCL and one (*N* = 1) due to GVHD.

**Conclusion:** Disease relapse after alloHSCT can be controlled with available treatments. For most patients who ultimately relapsed, reduction of immunosuppression and interferon alpha−2a either administered alone or in combination with another systemic agent were preferred. Although interferon alpha−2a, similarly to immunosuppression reduction, may be beneficial for the achievement of graft–vs.–lymphoma effect, the risk of simultaneous worsening of GVHD must be carefully evaluated and taken into consideration.

## Introduction

Primary cutaneous T–cell lymphomas (CTCL) are a heterogeneous group of non–Hodgkin lymphomas (NHL) of skin—homing T–cells (1, 2). CTCL account for ~2% of all lymphomas and mycosis fungoides (MF) comprise the majority of cases. Most patients with MF present a prolonged, indolent clinical course with initial skin involvement and subsequent progression in certain cases to the lymph nodes and visceral organs (3, 4). Early disease stages can be controlled with skin—directed therapy, such as topical steroids, light treatment and radiation (5). Sézary syndrome (SS) is a rare (2–5%), aggressive leukemic variant of CTCL with systemic features in addition to skin involvement, characterized by low complete response (CR) rates to therapy (3, 4). Advanced MF and SS have a dismal prognosis and warrant systemic therapy; yet, long—term remission rates with conventional treatments alone are still low (5).

Hematopoietic stem cell transplantation (HSCT) has been explored as a curative option in patients with advanced—stage CTCL and has shown promising disease control (DCR) and overall survival (OS) rates (6, 7). A patient—level meta—analysis has implied that autologous HSCT results only in limited responses with OS and progression—free survival (PFS) inferior to allogeneic HSCT; therefore, autologous HSCT in CTCL is currently barely used (8). Several sets of clinical data have indicated that allogeneic HSCT could provide a cure in patients with previously incurable disease (7). Although randomized clinical trials comparing HSCT and conventional systemic therapies are missing, OS rates after HSCT are encouraging and are estimated at 46 and 44% at 5 and 7 years, respectively (9–11). Nevertheless, about 50% of the patients will relapse during the first year post—HSCT, with a median time of disease relapse at 3.8 months (9). Although most cases describe local, manageable skin relapse, hematological relapse has been associated with a poor prognosis (9). To date, there is yet no established consensus for the management of disease relapse after HSCT. Herein, we report the results of a single center, retrospective analysis of ten CTCL patients treated with alloHSCT with a long—term follow—up and we aim to discuss possible therapeutic strategies for the management of disease relapse after alloHSCT.

## Methods

Medical records of 215 patients diagnosed with CTCL in the Dermatology clinic of the University Hospital of Zurich between 2012 and 2019 with a closing date of February 2020 were reviewed. Clinical data of patients who underwent HSCT were retrospectively collected. Two patients (*N* = 2) who underwent an autologous HSCT were excluded. In total, ten (*N* = 10) patients with advanced—stage CTCL (stages IIB and higher) who underwent a first alloHSCT were identified and further analyzed. Minimum follow—up after HSCT was set to 3 months. Baseline demographic characteristics, disease and transplantation characteristics were collected through their medical records. Diagnosis was based on local clinic and histologic review. TNM classification was adopted according to the International Society for Cutaneous Lymphomas (ISCL) and the European Organization of Research and Treatment of Cancer (EORTC) (1, 12). Data analysis focused on disease outcome, including progression—free survival (PFS, defined as date of HSCT to disease progression), time to next treatment (TTNT, defined as stop date of HSCT to date of next treatment initiation) and overall survival (OS, defined as time from HSCT start until last visit or death). End points were assessed on the date of last patient contact. Follow—up time was calculated from the start date of alloHSCT to the date of last follow-up, including last visit or date of death, or February 2020, whichever occurred first. Patients alive at the end of follow—up were censored. All analyses were conducted using statistical language R version 3.5. Reported *p*-values were accepted as statistically significant if <0.05.

Written informed consent for retrospective analysis of CTCL patients was approved by Zurich ethics committee (KEK-ZH 2014-0193).

## Results

### Patient Characteristics

Clinical characteristics are summarized in Table 1. A total of ten patients with CTCL and median age of 56.5 years (range 22–66) were included. Seven patients were males (7/10) and three were females (3/10). CTCL type included mycosis fungoides and folliculotropic mycosis fungoides (MF and FMF, *N* = 7), Sézary syndrome (SS, *N* = 1), extranodal EBV+ NK/T–cell lymphoma, nasal type (NNKTL, *N* = 1) and aggressive epidermotropic cytotoxic T-cell lymphoma (AECTCL) as composite lymphoma with chronic lymphocytic leukemia (CLL) (*N* = 1). Three patients (*N* = 3) diagnosed with FMF had an advanced disease with histologically follicle—based infiltrated tumors, which is associated with an aggressive course and dismal prognosis (13). Large cell transformation (LCT) was present in one of these patients (1/3, patient Nr. 8). Staging information during the initial CTCL diagnosis is summarized on Supplementary Table 1, available at *Frontiers in Medicine supplement*.

**Table 1:** Baseline characteristics and alloHSCT characteristics (*N* = 10).

Age at diagnosis, years (median, range)	56.5 (22–66)
Sex, *n*	
Male	7
Female	3
Initial Staging, *n*	
IB	5
IIB	2
IIIA	1
T1bN0M0	1
T3bN3M1	1
Initial treatment, *n*	
PUVA	7
MTX	4
IFN–alpha	8
Retinoids	5
Mogamulizumab	1
Brentuximab–vedotin	3
Chemotherapy	8
Best overall response to initial treatment, *n*	
CR	2
PR	7
PD	1
Total lines of therapy before alloHSCT, excluding conditioning regimen (median, range)	4 (1–5)
Stage at alloHSCT, *n*	
IIB	3
IVA2	5
IVB	2
T2bN2M0	1
T3bN3M1	1
Remission status before alloHSCT, *n*	
CR	1
PR	5
PD	4
Conditioning regimen	10
Busulfan, fludarabine and anti–thymocyte globulin (ATG)	9
Busulfan, fludarabine and thiotepa	1
Donor type, *n*	
Matched sibling	3
Matched unrelated	7
GVHD prophylaxis, *n*	
Cyclosporine and MMF	9
Ciclosporine, MMF, Cyclophosphamide	1
Disease relapse post–alloHSCT, *n*	7
Time of alloHSCT to first relapse, months (median, range)	3.3 (0.5–7.4)
Time to next treatment, months (median, range)	8.1 (1.9–9.4)
GVHD Grade, *n*	8
I	1
II	4
III	1
IV	2
Course, *n*	
Alive	6
Dead	4
Time of alloHSCT to last visit or death, months (median, range)	12.6 (3.5–73.7)

*PUVA, psoralen and ultraviolet A; MTX, methotrexate; CR, complete response; PR, partial response; PD, progressive disease; alloHSCT, allogeneic hematopoietic stem cell transplantation; GVHD, graft vs. host disease; MMF, mycophenolate mofetil*.

In total, eight patients had received three or more lines of systemic therapy prior to HSCT. Excluding HSCT and conditioning regimen, the median number of treatment regimens received was 4 (range 1- 5). Treatments included PUVA (*N* = 7), methotrexate (MTX, *N* = 4), retinoids (*N* = 5), IFN—alpha (*N* = 8) and mogamulizumab (anti-CCR4 monoclonal antibody, *N* = 1) in terms of a clinical trial (NCT01728805). Three patients with CD30+ MF received brentuximab—vedotin (anti-CD30 monoclonal antibody, *N* = 3) in terms of a clinical trial (NCT01578499). Eight patients (*N* = 8) received chemotherapy as tumor debulking therapy prior to HSCT, including CHOP, R–CHOP plus etoposide, doxorubicin, pralatrexat, vorinostat, gemcitabine, asparaginase, ifosfamide and DHAP (dexamethasone, high-dose AraC, platinol). Complete remission (CR) of disease was achieved in only one patient prior to alloHSCT (*N* = 1). Five patients (*N* = 5) were in partial remission (PR), whereas four patients (*N* = 4) experienced disease progression (PD).

### Allogeneic Hematopoietic Stem Cell Transplantation (alloHSCT)

AlloHSCT characteristics are summarized in Table 1. In all, ten patients (*N* = 10) underwent allogeneic hematopoietic stem cell transplantion. The vast majority of the patients received a reduced—intensity conditioning (RIC) regimen including busulfan, fludarabine and anti—thymocyte globulin (ATG) (*N* = 9) whereas one patient (*N* = 1) received busulfan, fludarabine and thiotepa. Donor type for alloHSCT was matched unrelated in seven patients and matched related in three patients. CR was achieved in eight patients after alloHSCT. One patient with NNKTL (*N* = 1) and one with FMF (*N* = 1) did not respond to the alloHSCT (PD). The overall response rate (ORR) at month 3 after HSCT was 80% (CR, *N* = 7 and PR, *N* = 1); during this time, disease relapse occurred in one patient with FMF at 58 days post–HSCT. As of February 2020, three patients (30%) remained free – of disease relapse and six patients (60%) were alive since alloHSCT.

### Graft vs. Host Disease (GVHD)

GVHD prophylaxis consisted of cyclosporine and mycophenolate mofetil (MMF) in nine alloHSCT patients (*N* = 9). One patient (*N* = 1) received GVHD prophylaxis with cyclosporine, MMF and cyclophosphamide. Acute GVHD developed in eight of ten patients (grade 1, *N* = 1; grade 2, *N* = 4; grade 3, N =1; grade 4, *N* = 2). One patient with NNKTL deceased due to grade IV GVHD with cutaneous, pulmonary and gastrointestinal manifestation, 14.6 months after alloHSCT.

### Disease Progression, Relapse and Outcome

Seven patients (*N* = 7) relapsed after a median time of 3.3 months (0.5–7.4 months). Four out of seven (4/7) patients who experienced disease—relapse received further systemic treatment, three of which (3/7) in combination with skin—directed treatment for cutaneous relapse (radiotherapy (RT), operation). Systemic treatments included adoptive T–cell transfer with EBV specific cells (*N* = 1) interferon alpha-2a combined either with donor lymphocyte infusion (*N* = 1) or with brentuximab—vedotin (*N* = 1) and R-CHOP in one (*N* = 1) patient with AECTCL due to rapid disease progression post—alloHSCT. One patient (1/7) with SS received only RT for cutaneous disease and in one (1/7) patient, reduction of immunosuppression without any subsequent treatment resulted in disease control. Systemic treatment was not considered in one patient (1/7) due to reduced performance status.

Two patients (Nr. 1 and 2, presented on Supplementary Table 1), each experienced two disease relapses. The first patient (Nr. 1) initially relapsed 7.4 months after alloHSCT and received a skin—directed treatment with radiotherapy. During the second relapse 9.6 months post—alloHSCT, a systemic treatment with interferon alpha−2a was initiated with subsequent CR. The second patient (Nr. 2) initially relapsed 6 months after alloHSCT and was treated with interferon alpha−2a in combination with donor lymphocytes. The second relapse occurred after 15.1 months post-alloHSCT and treated with brentuximab—vedotin with subsequent PR. Median TTNT was 8.1 months (1.9–9.4 months). ORR was 57.1% (CR, N =2; PR, *N* = 2).

**Figure 1:**
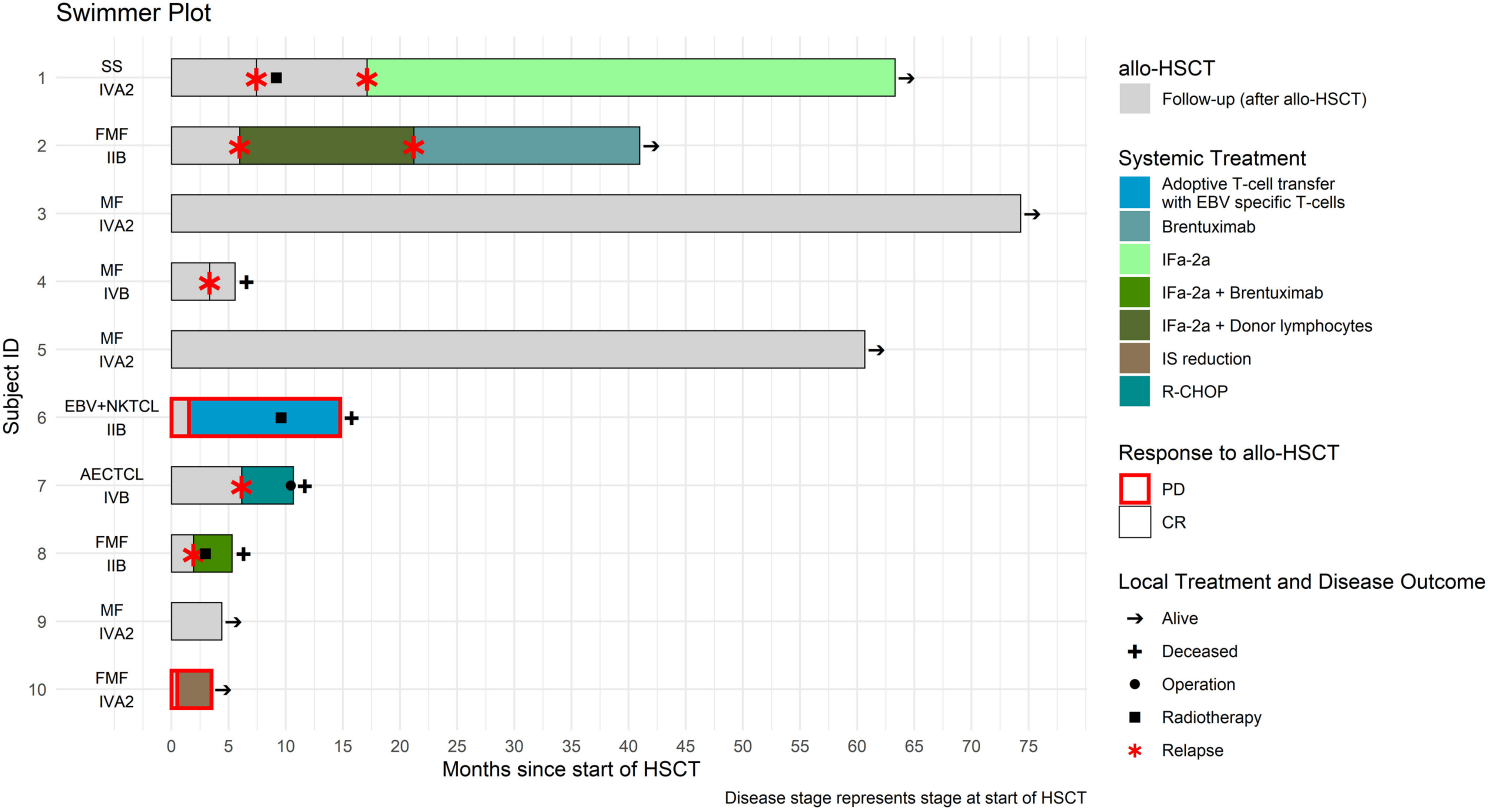
Treatment course of the ten patients who underwent allogeneic hematopoietic stem cell transplantation (alloHSCT).

After a median follow-up time of 12.6 months (3.5–73.7 months) post—alloHSCT, six patients were alive (60%) and four had deceased, three (*N* = 3) due to CTCL progression and one (*N* = 1) due to GVHD. Treatment course and follow—up data for the all patients who underwent HSCT are summarized in Figure 1 and Supplementary Table 1 available at *Frontiers in Medicine supplement*.

### Overall Survival (OS) and Progression—Free Survival (PFS)

**Figure 2:**
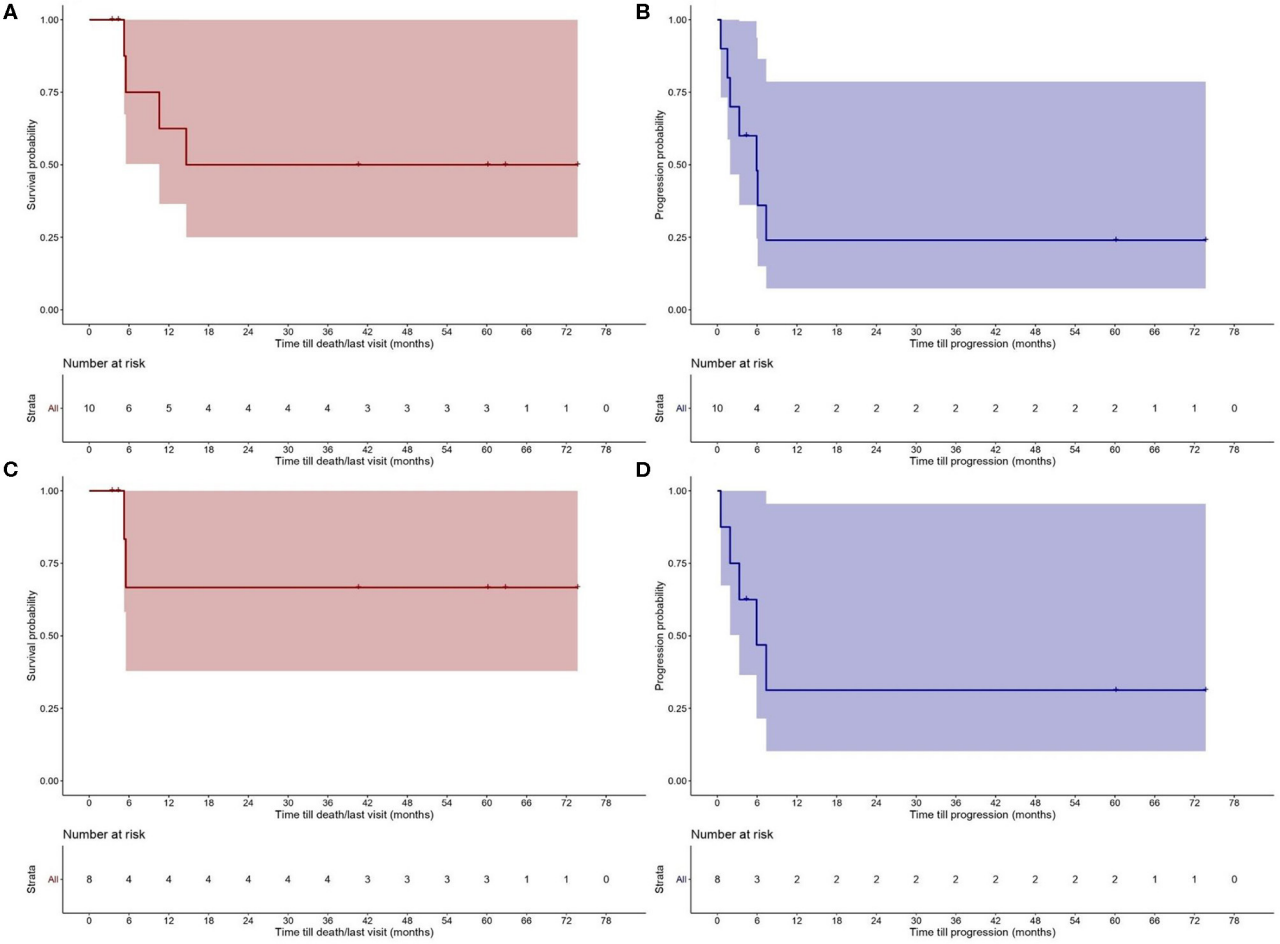
Probability of **(A)** overall survival (OS) and **(B)** progression—free survival (PFS) after allogeneic hematopoietic stem cell transplantation (alloHSCT) for the study population. OS **(C)** and PFS **(D)** after alloHSCT for patients with Sézary syndrome (SS), mycosis fungoides (MF) and folliculotropic mycosis fungoides (FMF).

**Figure 3:**
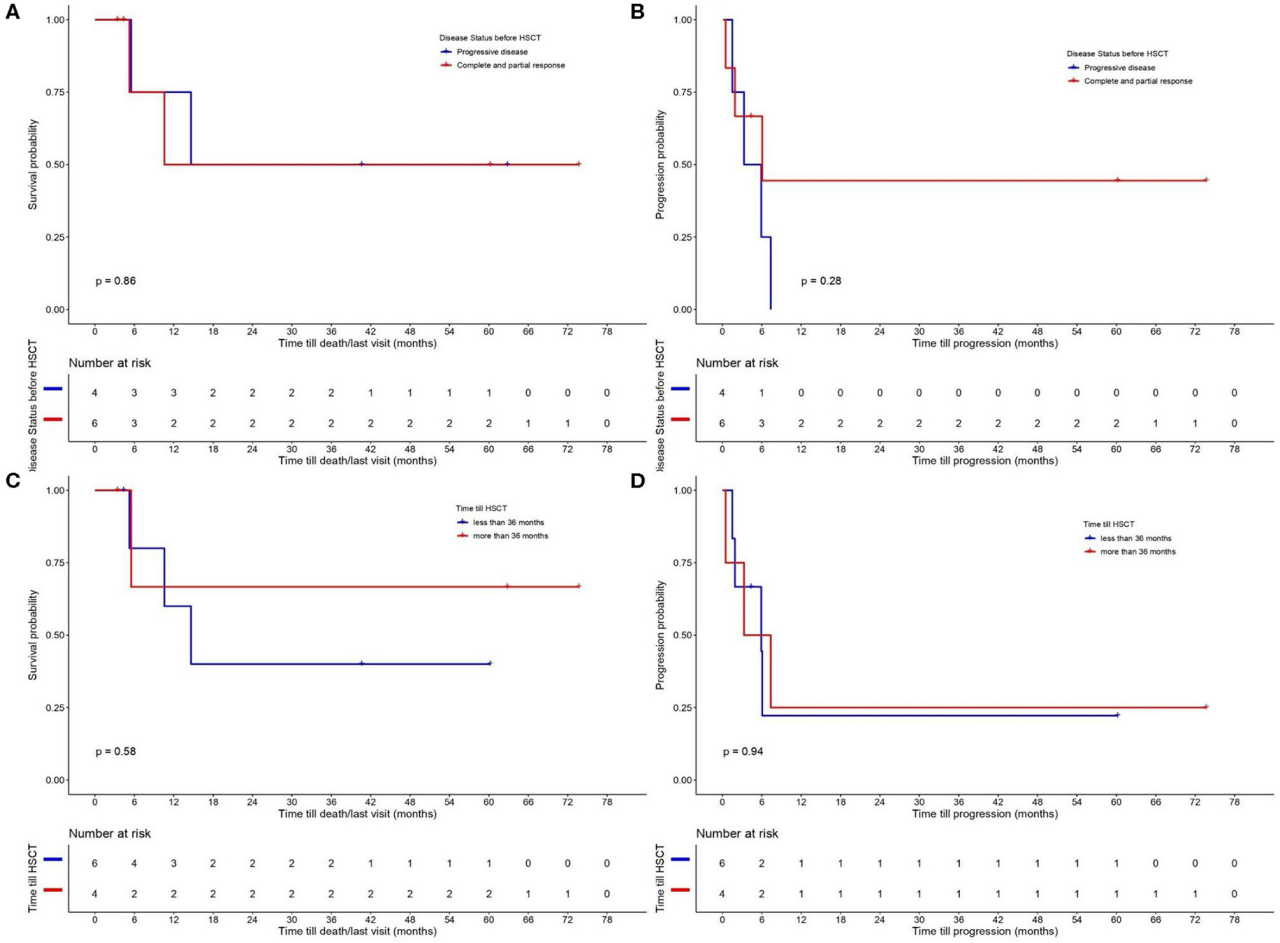
Probability of **(A)** overall survival (OS) and **(B)** progression—free survival (PFS) according to disease status prior to allogeneic hematopoietic stem cell transplantation (alloHSCT) (comparing recipients with minimal residual disease (CR or PR) with patients on PD before HSCT). OS **(C)** and PFS **(D)** according to time till alloHSCT with a cut–off 36 months from disease diagnosis.

The results for OS and PFS for all CTCL types are provided in Figure 2. PFS was 25% at 12 months and OS 62.5 and 50% at 12 and 24 months, respectively. A separate analysis was performed to evaluate the disease control and outcome after alloHSCT for patients diagnosed with MF, FMF, and SS, due to the different nature of the two other lymphomas (NNKTL and AECTCL) and their possible impact on PFS and OS. PFS for this patient group was 30% at 12 months and OS 65% at 6, 12 and 24 months (Figure 2). We further analyzed the OS and PFS rates according to the disease status prior to transplant (Figures 3A,B) and timing of transplant (Figures 3C,D), with a cut—off 36 months from disease diagnosis. We found no benefit on PFS and OS on “earlier” transplants (<36 months after disease diagnosis) compared to “later” transplants (*p* = 0.94 and *p* = 0.58, respectively). Concerning the disease status prior to transplant, we compared recipients with minimal residual disease (CR or PR) before HSCT with patients having PD. There was a statistically non-significant difference on PFS (*p* = 0.28) in favor of CR/PR compared to PD prior to HSCT. Though, no difference on OS was found (*p* = 0.86), which can be explained by the systemic treatment initiation after disease relapse. Indeed, only one patient (*N* = 1) with stage IVB MF and PD prior to HSCT deceased due to lymphoma.

## Discussion

According to the literature, about 70% of the CTCL cases, including mostly MF and SS, present with early disease stage (IA–IIA) and are associated with a favorable prognosis and 5–year OS survival rates of 96–99% (14–16). Patients with advanced disease stages have a poor prognosis and often warrant a systemic treatment (5). In a recent study, median OS for advanced, stage IIB, IVA and IVB MF and SS was 68, 48, and 33 months, respectively (17). Although diverse systemic treatment options are available, ORR with newer agents average 30–40% with scarce data concerning long—term disease control (17, 18). Current guidelines recommend alloHSCT as a second—line treatment option from stage IIB of MF (evidence level 3) and in SS (evidence level 3) for eligible patients to improve survival (18).

In this retrospective study, we describe the outcomes of ten patients with extensively pretreated, advanced CTCL who underwent alloHSCT in a single—center cohort. Since autoHSCT is no longer sought as a treatment modality in CTCL patients (8), these patients were excluded from the current analysis. The vast majority of the CTCL types included MF and SS. Three cases diagnosed with FMF had an advanced disease type, which is associated with an aggressive disease course and thus, dismal prognosis (13). Besides, rare entities with historically poor prognosis, such as NNKTL and AECTCL were also identified and included in the analysis (19). Due to the different nature of these two CTCL types compared to MF and SS, a separate analysis evaluating the PFS and OS rates only on MF/FMF and patients was performed. In the present study, the OS rate for the patient population was 62.5 and 50% at 1 and 2 years, respectively, and the PFS 25% at 1 and 2 years. Most patients seem to progress early after the HSCT; PFS at 5 months was 60% and 3 months after HSCT ORR was 80%, with seven patients reaching CR. For patients diagnosed with either MF/FMF or SS, PFS was 30% at 12 months and OS 65% at 6 months, with sustainable responses at 12, 18, and 24 months post—HSCT. Although a direct comparison with previously reported studies is difficult and could be misleading, these sustainable responses seem to be similar to those described for alloHSCT (20, 21). Extended analysis from retrospective published data reported OS of 46 and 44% at 5 and 7 years for alloHSCT in patients with MF or SS (22).

In our patient cohort, as of February 2020 and with a median follow—up time 12.6 months (3.5–73.7 months), 60% of the patients were alive. Transplant—related mortality was low; out of the four patients that deceased, one patient developed stage IV GVHD (time from HSCT 14.6 months). Grade IV GVHD was observed in one patient with MF, though still alive at the data closing date. GVHD rates of varying severity grades were observed in eight out of ten patients who underwent an alloHSCT with three patients developing grade III and IV GVHD. De Masson at al suggested an antigenic stimulation by residual tumor cells as a possible mechanism of these high GVHD incidence rates in CTCL patients following alloHSCT (21). In this study, we did not observe any direct association of GVHD development with remission status before transplantation. Nevertheless, GVHD has been previously associated with prolonged PFS, presumably due to simultaneous graft—vs.—lymphoma (GVL) effect (23, 24).

A considerable number of patients in this study (*N* = 7) experienced disease relapse after a median time of 3.3 months (0.5–7.4 months), confirming previous data suggesting that the majority of relapses occur within the first year post—transplant. Most patients who experienced relapse post—HSCT were induced back into remission by skin—directed or systemic treatment, as well as reduction of immunosuppression. Systemic treatment was not considered in one patient due to a reduced performance status. In the vast majority of the patients who relapsed (*N* = 3), reduction of immunosuppression and systemic treatment with IFN—alpha in combination with donor lymphocytes and brentuximab vedotin was preferred, attempting to increase the antitumor effect. Indeed, some cases have currently provided clinical evidence of a GVL effect in patients with MF/SS (23, 25, 26), supporting that immunosuppression reduction and IFN—alpha may promote an immune-mediated GVL effect in patients who underwent an alloHSCT. Preclinical data on mouse models confirm that type 1 interferons enhance protective GVL responses through donor cell production of IFNα/β, thus providing experimental support for IFN—alpha as a treatment option in individuals at high risk of relapse after HSCT (27). Nevertheless, the risk of simultaneous induction and/or worsening of post-transplant GvHD with severe consequences must be carefully evaluated and taken into clinical consideration.

The timing of HSCT and choice of conditioning regimen are subject to controversy. In our patient cohort, RIC with busulfan, fludarabine and anti—thymocyte globulin (ATG) was previously used in the majority of the patients who underwent an alloHSCT to allow treatment in patients with comorbidities, multiple previous treatment—lines and advanced disease stage. Although the ideal conditioning regimen for HSCT in CTCL patients is unknown, recent studies suggest that RIC reduces the mortality rates seen with myeloablative conditioning regimen (MAC) with equivocal efficacy and should be therefore recommended in eligible patients undergoing alloHSCT (11, 20, 28). A less intensive approach with non-myeloablative preparative regimen using total lymphoid irradiation (TLI) and anti-thymocyte globulin (ATG), without chemotherapy, is currently investigated in a phase−2 clinical trial (NCT00896493) with promising preliminary results (29).

In this study, we could not find any significant difference between “earlier” (<36 months after disease diagnosis) compared to “later” transplants on OS and PFS. However, this may be explained by the small patient cohort. Retrospective studies on larger patient cohorts have linked worse OS, reduced PFS and increased risk of disease relapse to advanced stage of disease at the time of HSCT (11, 22). Although the optimal timing for HSCT has not been yet determined, we recommend early consultation of a transplant physician in transplant eligible patients, in line with the EORTC recommendations (18).

A main strength of our study is the detailed clinical data and follow—up information available for CTCL patients, including those treated with HSCT at our institution. However, the small number of patients, the retrospective nature of the data and the heterogeneity of CTCL are possible limitations of this analysis. Given the rarity of CTCL and the high—quality follow—up data on HSCT in CTCL, our study provides interesting conclusions on the effect of HSCT in CTCL and the management of relapses from a single center. We conclude that alloHSCT may induce durable responses and should be therefore considered as a treatment option for patients with advanced or treatment—refractory CTCL, especially for patients with more aggressive CTCL subtypes, such as advanced stage folliculotropic MF or presence of large cell transformation. In accordance with previous studies (29) and with the current guidelines (18), we recommend alloHSCT for patients with MF/SS with advanced disease stages (IIB to IV) who are in first or second CR, PR or relapse/progression having received three or fewer prior lines of systemic therapy. Regarding the conditioning regimen, Duarte et al. showed that using RIC regimens decreased the non-relapse mortality (NRM) significantly without increasing the risk of relapse (9). This led to a better OS, also in younger patients. The RIC we routinely use includes fludarabin, busulfan and ATG (for unrelated and related donors). Furthermore, there are many case reports which provide evidence of a graft—vs.–tumor effect in patients with MF/SS. Duarte al reported that 50% patients with a relapse after alloHSCT achieve a CR after receiving donor lymphocyte Infusion (DLI) (9). However, there is too little data to recommend prophylactic DLI in this patient group. We recommend that disease relapse post-alloHSCTshould be promptly treated with reduction of immunosuppression and immunomodulatory agents, in order to control relapse and to enhance graft—vs.—lymphoma effect. However, further improvements to control the severity of GVHD need to be pursued.

## Data Availability Statement

The datasets generated for this study are available on request to the corresponding author.

## Author Contributions

FD and EG: study concepts, study design, data acquisition, data analysis and interpretation. FD and SK: statistical analysis. FD: article preparation. FD, US, GN, SK, M-CB, WH, LF, RD, AC, and EG: article editing and review. All authors contributed to the article and approved the submitted version.

## Conflict of Interest

FD receives intermittent travel support from Pierre Fabre outside of the submitted work. RD has intermittent, project focused consulting and/or advisory relationships with Novartis, Merck Sharp & Dhome (MSD), Bristol-Myers Squibb (BMS), Roche, Amgen, Takeda, Pierre Fabre, Sun Pharma, Sanofi outside the submitted work. EG has intermittent, project focused consulting and/or advisory relationships with Mallinckrodt, Takeda, Helsinn and Novartis, outside the submitted work. The remaining authors declare that the research was conducted in the absence of any commercial or financial relationships that could be construed as a potential conflict of interest.
