# Supplementary material for: Long–Term Disease Control After Allogeneic Hematopoietic Stem Cell Transplantation in Primary Cutaneous T–Cell Lymphoma; Results From a Single Institution Analysis
Source: Front Med (Lausanne). 2020 Jun 25;7:290. doi: 10.3389/fmed.2020.00290 (PMC7344271; doi:10.3389/fmed.2020.00290)
Supplement: Supplementary file 1 [file Table_1.docx]

**SUPPLEMENTAL ONLINE CONTENT**

**Supplemental tables:**

**eTable 1.** Patient characteristics and follow – up data (N =10). CTCL: cutaneous T – cell lymphoma, SS: Sézary syndrome, MF: Mycosis fungoides, FMF: folliculotropic mycosis fungoides, NNKTL: extranodal NK/T – cell lymphoma, nasal type, AECTCL: aggressive epidermotropic cytotoxic T-cell lymphoma, CLL: chronic lymphocytic leukemia, ORR3: overall response ate month 3, HSCT: allogeneic stem cell transplantation, CR: complete response, PR: partial response, PD: progressive disease, GVHD: graft versus host disease, IS: immunosuppression

**eTable 1.**

| **Age (at first diagnosis)/Sex** | **CTCL Type** | **Year of diagnosis** | **Initial staging** | **Remission status before HSCT** | **Stage at HSCT** | **Type of HSCT** | **Course** | **ORR3** | **First Relapse** | **Systemic treatment after first relapse** | **Second relapse** | **Systemic treatment after second relapse** | **Course** |
| --- | --- | --- | --- | --- | --- | --- | --- | --- | --- | --- | --- | --- | --- |
| 58/M | SS | 2009 | IB | PD | IVA2 | Allogeneic | CR | CR | yes | no (IS reduction) | yes | Interferon alpha-2α | Alive |
| 66/M | FMF^1^ | 2013 | IB | PD | IIB | Allogeneic | CR | CR | yes | Interferon alpha-2α, Donor lymphocytes | yes | Brentuximab vedotin | Alive |
| 25/M | MF | 2001 | IB | PR | IVA2 | Allogeneic | CR | CR | no |  |  |  | Alive |
| 22/M | MF | 1994 | IB | PD | IVB | Allogeneic | CR | CR | yes | no | no |  | Dead (CTCL) |
| 57/W | MF | 2013 | IIIA | PR | IVA2 | Allogeneic | CR | CR | no |  |  |  | Alive |
| 61/W | NNKTL | 2015 | T1bN0M0 | PD | T2bN2M0 | Allogeneic | PD | PD | yes | Adoptive T-cell transfer with EBV specific T-cells | no |  | Dead (GVHD) |
| 56/M | AECTCL | 2016 | T3bN3M1 | CR | T3bN3M1 | Allogeneic | CR | CR | yes | R-CHOP | no |  | Dead (CTCL) |
| 61/M | FMF^1^ | 2016 | IIB | PR | IIB | Allogeneic | CR | PD | yes | Interferon alpha-2α, Brentuximab vedotin | no |  | Dead (CTCL) |
| 49/M | MF | 2017 | IVA2 | PR | IVA2 | Allogeneic | CR | CR | no |  |  |  | Alive |
| 51/F | FMF^1^ | 2015 | IIB | PR | IVA2 | Allogeneic | PD | PR | yes | no (IS reduction) | no |  | Alive |

1. Corresponds to an advanced FMF stage with histologically follicle – based infiltrated tumors. Large cell transformation (LCT) was present in patient Nr. 8.
